# Process Evaluation of Animal-Assisted Therapy: Feasibility and Relevance of a Dog-Assisted Therapy Program in Adults with Autism Spectrum Disorder

**DOI:** 10.3390/ani9121103

**Published:** 2019-12-09

**Authors:** Carolien Wijker, Ruslan Leontjevas, Annelies Spek, Marie-Jose Enders-Slegers

**Affiliations:** 1 GGZ Oost Brabant, P.O. Box 3, 5427 ZG Boekel, The Netherlands; 2 Faculty of Psychology and Educational Sciences, Open University, Valkenburgerweg 177, 6419 AT Heerlen, The Netherlands; roeslan.leontjevas@ou.nl (R.L.); marie-jose.enders@ou.nl (M.-J.E.-S.); 3 Department of Primary and Community Care, Center for Family Medicine, Geriatric Care and Public Health, Radboud University Nijmegen, Medical Centre, Geert Grooteplein Zuid 10, 6525 GA Nijmegen, The Netherlands; 4 Autism Center of Expertise, Goyergracht Zuid 39, 3755 MZ Eemnes, The Netherlands; anneliesspek@hotmail.com

**Keywords:** autism, adults, animal-assisted therapy, dogs, feasibility

## Abstract

**Simple Summary:**

An explorative study on the effects of animal-assisted therapy showed reduced stress and improved social communication in adults with autism spectrum disorder (ASD). To examine whether this intervention is suitable for a broader scope of application in clinical practice, we conducted a process evaluation alongside the effect study. The aim of this process evaluation was to gain insight into the quality of the study, the relevance and feasibility of the intervention, and the barriers and facilitators to the implementation of the intervention. Questionnaires, semi-structured interviews, and treatment reports were used to analyze process data in 27 participants who were randomized into the intervention condition and in three therapists. Both the participants and therapists evaluated the animal-assisted therapy program as satisfying, feasible, and relevant for adults with ASD. They reported the following arguments for their positive appraisal of the therapy: the therapy helped improve self-insight, joy, relaxation, and physical contact. The participants’ attitudes, personal skills for generalization, and severity of contextual problems were named as potential barriers to the feasibility of the therapy program. Females and dog owners were over-represented in this study, and therefore, generalization of the previously established effects should be made with caution. However, given the intervention’s effects and the positive evaluation of the program by the participants and therapists alike, animal-assisted therapy can be considered a valuable addition to treatment possibilities for reducing stress and improving social communication in adults with autism spectrum disorder.

**Abstract:**

(1) Background: Randomized controlled trials (RCTs) are considered the gold standard for evaluating a treatment. However, the results of an RCT may remain meaningless for clinical practice in cases of poor intervention feasibility or fidelity (the extent to which the protocol was executed), or when health care professionals or patients experience the intervention as irrelevant or unpleasant. Feasibility and relevance of psychosocial interventions are highly understudied in adults with autism spectrum disorder (ASD). In order to put the effects revealed in an RCT on an animal-assisted therapy (AAT) program in adults with autism spectrum disorder (ASD) into the context of clinical practice and to formulate guidelines for potential improvements and further implementation of the therapy, the aim of this process evaluation was to gain insight into the relevance and feasibility of the intervention and barriers and facilitators to its implementation. (2) Methods: Data were collected from 27 participants with ASD and three therapists using questionnaires, semi-structured interviews, and treatment reports. Reach, adherence, program fidelity, and program appraisal were evaluated, and barriers and facilitators to recruitment and implementation of the AAT program were explored. (3) Results: The participants were satisfied with the program and evaluated it as feasible and relevant for adults with ASD. The participants documented improving self-insight, joy, relaxation, and physical contact with a therapy dog as the reason of their positive appraisal of the therapy. Documented aspects that may influence feasibility and appraised relevance were the participants’ therapy attitude, skills for generalization, and severity of contextual problems (e.g., problems at work, relationship problems). Regarding the sample quality, females and dog owners were slightly over-represented in the RCT. (4) Discussion: Considering the positive evaluation of the intervention and its positive effects revealed in the RCT, the AAT program can be added to the treatment repertoire to reduce stress and improve social communication in adults with ASD. More research in larger samples is needed for better understanding the generalization of the intervention effects, especially in male patients and those who do not have a dog at home.

## 1. Introduction

The current paper describes a process evaluation of a randomized controlled trial (RCT) on the effectiveness of dog-assisted therapy aimed at reducing psychosocial problems, such as stress, depression, and anxiety, and improving social communication and self-esteem in adults with autism spectrum disorder (ASD) [1]. Although adults with ASD show high levels of comorbid problems [2,3], psychosocial interventions have been highly understudied [4], and effective treatments remain limited for these patients.

The RCT is considered the gold standard for providing evidence on the effectiveness of a therapy [5]. However, poor internal and external validity might render the RCT results meaningless for clinical practice. For example, poor internal validity may be the result of inadequate screening, recruitment, or randomization procedures (further addressed as sampling quality). This may also result in a non-representative study sample that undermines the external validity of the RCT results. Furthermore, both internal and external validity can be compromised when not all of the intervention elements are executed properly in an RCT. The study results should, then, be attributed to only several elements of the intervention, rather than the whole intervention. When therapists and patients are not satisfied with the intervention (e.g., due to severe side effects in patients and procedures that are not clear for the therapists) or when they find the intervention irrelevant for the target population, the poor intervention quality experienced may result in poor implementation in health care practice.

A process evaluation provides insight into the aspects that determine the internal and external validity of an RCT. It can also reveal potential barriers and facilitators to implementing an intervention [6]. This information is important for health professionals and stakeholders for both interpretation of study results and choosing the most suitable and effective interventions for clinical practice. Furthermore, via thorough process evaluations, rich and specific data can be collected and reported, which provide a good understanding of the variables involved in the relationship between the intervention and study outcomes in the RCT. This information can help understand why an intervention has worked, or why not, and can contribute to theoretical discussions about its working mechanisms. In the past decades, the importance of a process evaluation alongside an RCT has been acknowledged in medical and health care research. For example, guidelines have been published to establish an adequate process evaluation [7], and the growing number of process evaluation reports has even resulted in systematic reviews on process evaluations to provide information for refining existing and developing new interventions [8,9].

To the best of our knowledge, besides our RCT on dog-assisted therapy [10], the literature regarding adults with ASD describes only two non-pharmacological interventions. These are cognitive behavioral therapy (CBT) and mindfulness-based stress reduction (MBSR). Both interventions showed favorable effects on comorbid problems, such as anxiety [11]. Although no process evaluations were reported, several adjustments of the original intervention protocols were recommended to increase the feasibility of the interventions [4]. For example, Spek et al. adjusted the MBSR protocol of Segal, Williams, and Teasdale (2002) [12] to make it suitable for the ASD target group [13]. Because adults with ASD may have information processing problems, Spek et al. proposed avoiding metaphors and exercises in which the participant observes his or her own thoughts. In general, adults with ASD have difficulties in communication, representation, information processing, and generalization of learned techniques into daily life [14,15]. Such difficulties may pose real challenges for a strict execution of an intervention protocol, resulting in poor program fidelity (the extent to which the intervention is delivered in accordance with the protocols) [16]. However, even excellent feasibility during an RCT does not guarantee that the feasibility of the intervention will be suitable in clinical practice. Different external factors (e.g., financial aspects, such as insurance coverage) can jeopardize program fidelity of the total intervention or its specific components. Moreover, factors related to the ASD population can influence the feasibility of an intervention and the generalizability of previously reported effects. For example, patients with ASD may experience difficulties in representation and imagination of a future perspective. Therefore, patients with ASD may not be motivated to execute exercises that do not lead to immediate results or changes in their daily lives.

Animal-assisted therapy (AAT) in patients with ASD is an intervention that includes both a trained animal and pre-established therapeutic goals guided by a therapist certified in health care [17]. In children with ASD, positive effects were found on social impairments, social communication, and stress reduction after an animal-assisted intervention (AAI) [18,19]. To the best of our knowledge, to date, our study is the only AAI study that has been conducted in adults with ASD. The results showed decreased self-reported stress and agoraphobia and improved proxy-reported social awareness and social communication after a ten-week dog-assisted therapy program [10]. Although these effects look promising, health professionals and researchers also need information on aspects that can affect internal and external validity, such as sampling quality, program fidelity, and appraisal of the therapy program by the patients and health care professionals. Alongside estimated effects of an intervention, information on potential barriers and facilitators to AAT is needed to help research consumers (e.g., professionals in clinical practice and decisionmakers involved in financing care) make informed decisions.

The aim of this process evaluation was to investigate study quality and program relevance and feasibility of an AAT program tested in adults with ASD and to gain insight into barriers and facilitators to implementing the intervention within health care organizations. The results of this process evaluation can help place the study effects in context and provide guidelines to facilitate the transition from research evidence to clinical health practice.

## 2. Materials and Methods 

### 2.1. Design

The process evaluation described in this paper was executed alongside the RCT conducted at the mental health care organization GGZ Oost Brabant, the Netherlands [10]. This organization has a psychiatric outpatient center for adults with ASD with normal to high intelligence. Because the RCT was the first study on the effectiveness of AAT in adults with ASD, the study had an exploratory character. It aimed to include 72 participants, a comparable sample size to other intervention studies in adults with ASD [13,20,21]. The participants were recruited sequentially in batches from the mental health care organization and the Dutch Society for Autism (Nederlandse Vereniging voor Autisme, NVA) through information flyers placed in the waiting room and via verbal information from their therapist. After recruiting and screening for eligibility, a baseline assessment (T0) was conducted, and the participants were randomized into one of two conditions (intervention vs. waiting list control). At 10 and 20 weeks after the baseline, post treatment (T1) and a follow-up measurements (T2) were conducted. After T2, the participants in the waiting list control group were given the possibility of receiving AAT. Detailed information about methods can be found in the study protocol [1]. The medical ethics committee CMO region Arnhem-Nijmegen, the Netherlands, approved the study NL48974.091.14.

### 2.2. Intervention

In this intervention, AAT was provided by a certified professional with a college or university degree in psychology who was specialized in working with adults with ASD. All the therapists had a minimum of five years of working experience with this target group. The program incorporated trained service dogs (*N* = 13) from the Dutch service dog foundation (Stichting Hulphond Nederland) and included the following: Labradors (*N* = 2), Labrador crossbreeds (*N* = 4), Poodles (*N* = 2), a Golden Retriever (*N* = 1), Golden Retriever crossbreeds (*N* = 3), and a German wirehaired pointer (*N* = 1). The therapy dogs were aged between 2 and 10 years and had been selected and trained by the Dutch Service Dog Foundation, which has formulated guidelines to protect and monitor animal welfare (e.g., hygiene and maximum working hours). Therapy dogs can work a maximum of 2 h per day (nonconsecutive) with a maximum of two days per week, depending on the breed, age, and fitness of the dog. The therapists also completed courses on dog behavior and monitored the stress of the dogs during the therapy sessions. In the participating mental health care organization, only three therapists working in the ASD-team had sufficient working experience with the ASD target group and met the requirements for working with therapy dogs. For that reason, these three therapists were selected to perform the therapy in this research.

It is noteworthy that concerning dog welfare and stress behaviors were documented in the participants’ treatment reports and were discussed with behavioral experts from the service dog foundation or, if necessary, with the veterinarian. No animals were harmed during the study.

### 2.3. Procedure

During the AAT sessions, the therapists used a semi-structured therapy protocol. The aim of the intervention was to reduce stress and stress-related outcomes, such as depression and anxiety and to improve social and communication skills. The intervention protocol consisted of 10 weekly sessions, each lasting 1 h. Detailed information about the AAT program has been described elsewhere [1]. Data regarding the process evaluation were obtained at T1 (10 weeks after the baseline). The participants and therapists were asked to fill in the process evaluation questionnaire (PEQ)—a questionnaire that covered the outcomes. Furthermore, after each session, a treatment report was documented by the therapist, and additional notes were made when (1) exercises were not or only partially completed, (2) a different therapy dog was involved in the sessions, and (3) when a different therapist (than the leading therapist) had taken over a session. In a process evaluation, it is important that the professionals who conduct the research and collect data are well informed about the content of the program that is being evaluated [7]. For this reason, a therapist who was not involved in the main effect study but was closely involved in the provision of the AAT program performed the semi-structured interviews on first- and second-order process data (e.g., sampling quality and barriers and facilitators to implementing AAT).

### 2.4. Outcomes

Two versions of a process evaluation questionnaire (PEQ) were used: a participant version (Appendix A) and a therapist version (Appendix B). Both PEQ versions contained questions about satisfaction, relevance, and feasibility. The participants could evaluate relevance and feasibility from two perspectives: from their own perspective and from that of others. A five-point scale from 1 = totally disagree to 5 = totally agree was used for responses. To evaluate the quality of the trial and implementation of the AAT program, first- and second-order process data were collected [11]. First-order data regarded sampling quality and intervention quality. Second-order data described barriers and facilitators to implementation of the AAT program.

### 2.5. First-Order Process Data

#### 2.5.1. Sampling Quality

Sampling quality was evaluated using the description of procedures for recruitment, informed consent, randomization, and data regarding barriers and facilitators to recruitment procedures. Reach was determined to assess which (sub)audience was included in the study. Age (divided into three groups (18–32), (33–46), and (47–60)), proportion of gender, and having a dog at home at T0 was descriptively compared with the proportions in the worldwide population [22,23].

Data on sampling quality were collected from the research database and from semi-structured interviews with the therapists involved in recruitment. Examples of questions in the semi-structured interviews include the following: ‘Can you name three reasons people mentioned for declination?’ ‘Do you have suggestions for recruitment?’

#### 2.5.2. Intervention Quality 

Intervention quality was evaluated using stakeholder feedback and by assessing adherence (the number of sessions completed by a participant) and feasibility (the extent to which the program elements were performed as intended).

Data on program feasibility were extracted from the treatment reports.

### 2.6. Second-Order Process Data

#### Barriers and Facilitators to Implementation

Data on barriers and facilitators to the implementation of the AAT program were collected using three open questions from the PEQ. Semi-structured phone interviews with the therapists were also performed to obtain further explanation and clarification of their responses on the PEQ (e.g., ‘What conditions do you think a therapy room should meet?’ and ‘Which exercises were, do you think, not feasible and why?’).

### 2.7. Data Analyses

Descriptive statistics (modi) were used for the quantitative data of the PEQ (SPSS version 21.0). The responses to the open-ended questions from the PEQ and semi-structured interviews were typed out by the principal researcher. Frequently reported concepts were categorized and labeled by the principal researcher and co-authors of this paper. Disagreements on the assignment of the categories were resolved by discussion.

## 3. Results

### 3.1. First-Order Process Data, Intervention, and RCT Quality

#### 3.1.1. Sampling Quality

*Recruitment and randomization.* The participants were recruited via their therapists at the mental health care organization GGZ Oost Brabant and information brochures in the waiting room, as well as through the Dutch Society for Autism, NVA. Study enrollment opened in January 2015 and remained open through July 2017. The study participants were recruited sequentially in batches (*N* = 7). Of the 169 individuals who were approached, 134 responded to the principal researcher and received a study information letter (*N* = 93 from the mental health care organization and *N* = 63 outside the organization (NVA)). A total of 51 individuals did not meet the inclusion criteria, and 50 individuals declined. Three main reasons for declining were reported as follows: (1) distance to the therapy location (*N* = 32; mainly individuals recruited via the NVA), (2) not able to stop with current treatment to participate in the study (*N* = 26), and (3) the intervention and measurement were considered too time consuming (*N* = 14). A total of 68 individuals (51%) responded to the information letter and were assessed for eligibility. Of the screened participants (*N* = 68), 8 individuals did not meet the inclusion criteria, and 7 individuals declined after assessment. A total of *N* = 53 screened participants met the inclusion criteria and provided verbal and written informed consent.

The participants within a batch were randomized blindly using computer-generated random numbers by the study’s methodologist (R. L.), who was not involved in recruitment or screening.

*Barriers and facilitators to recruitment.* Individuals who declined participation mainly reported the following reasons as barriers to recruitment: (1) distance to the location of the research and intervention, (2) health insurance: the participants had to register at GGZ Oost Brabant in order to receive the intervention, which was not possible when they were already receiving treatment for their ASD elsewhere, (3) concerns of both the therapists and participants about the additional burden of participating in this study (especially when the participants would be randomized into the waiting list control condition and would therefore not receive any intervention for 20 weeks), and (4) the exclusion criteria of the effect study: anxiety toward or allergy to dogs.

The principal researcher documented the following motivational determinants for recruitment: (1) awareness of the study by all the therapists in the autism department, (2) curiosity among the participants to learn more about an innovative intervention study, and (3) openness to contribute to research and to help other adults with ASD.

*Reach*. Reach was assessed as the proportion of priority audience or its subgroups that may participate in the intervention (Table 1).

The participants were divided almost equally over the three age groups within a range between 18 and 60 years old. Females and dog owners were over-represented in our study sample compared with the respectively estimated worldwide population and the Dutch population [22,23].

#### 3.1.2. Intervention Quality

Of all the participants (*N* = 53) included in the study, *N* = 27 were randomized into the intervention group and received the 10-week AAT program. *N* = 26 of the AAT participants responded to the PEQ questionnaire. Table 2 shows scores on the PEQ reported by the participants and therapists.

*Satisfaction.* The participants were satisfied with AAT. All the participants reported a score of 4 (satisfied) or 5 (very satisfied). In response to open questions, the participants mainly indicated positive experiences from AAT, such as joy, insight, reflection, and relaxation.

*Relevance and feasibility of AAT*. In general, the participants reported AAT as relevant and feasible for themselves and for other adults with ASD. The therapists reported the therapy as being very relevant and feasible. They also mentioned specific characteristics of AAT that underline their answers, such as concrete exercises that provide direct feedback on behavior; a safe and relaxing environment, which can create a lower threshold for participation; and the opportunity to touch another living being during the therapy sessions.

*Protocol adherence and program fidelity.* All the participants who were randomized into the intervention group (*N* = 27) received at least nine out of the 10 therapy sessions. A total of 22 (81%) participants received 10 therapy sessions, and five (19%) participants missed only one of the 10 sessions due to illness (*N* = 3) or vacation (*N* = 2).

In *N* = 16 participants (59%), several exercises prescribed by the therapy protocol were not performed or not performed to their full extent. The main reasons for deviation from the protocol (*N* = 51) are categorized and reported in Table 3.

The most commonly reported reason for deviation was “limited time” to complete all exercises in a session, for which the therapists reported different causes: (1) the slow processing speed of the participant, (2) the tendency of the participant to talk often, and (3) life events of the participant, which could not be ignored during therapy. One exercise (performing a self-made character: a role play exercise where the participant invents a character who interacts with the animal) was experienced as particularly difficult and therefore could not (completely) be performed by *N* = 8 (30%) participants.

Other reasons for making adjustments to exercises were as follows: the participant was already familiar with the exercise (*N* = 3, 11%); the temperature outside was above 25 °C, in which case, outdoor walking exercises were not performed out of consideration for dog welfare *N* = 2 (7%); the participant’s poor motor skills *N* = 2 (7%); and being overwhelmed by sensory stimuli from touching the dog *N* = 1 (4%).

Furthermore, the therapy protocol prescribes a fixed match between the therapy dog and participant. If a different therapy dog (e.g., a more playful one) seemed to offer better opportunities for a participant to reach therapy goals, a change was made in the dog–participant match in the sessions of *N* = 4 (15%) participants. Alternate therapy dogs were also used in sessions when (1) volunteers were not available to bring their therapy dog to the therapy location *N* = 5 (19%) or (2) the originally matched therapy dog was ill *N* = 3 (11%).

Reasons for a different therapist leading a specific therapy session included the therapist’s pre-planned holiday (*N* = 6 (22%) and therapist illness *N* = 3 (11%). For every participant, a different therapist led a therapy session for a maximum of one session.

### 3.2. Second-Order Process Data, Implementation

#### 3.2.1. Barriers to Implementing

The participants and therapists reported the following potential shortcomings in the basic requirements needed for an optimal AAT as barriers to implementing AAT in mental health care (Table 4): lack of eligible therapy dogs, lack of eligible therapists, and lack of suitable treatment rooms (too small, too much stimuli/noise, poor hygiene in therapy rooms, and slippery floors).

Furthermore, the participants and therapists described potential determinants that might jeopardize the feasibility of AAT, namely a negative attitude towards therapy in general (irrespective of AAT), stressful contextual factors in the participants’ lives, and problems with generalization.

#### 3.2.2. Facilitators to Implementing

The participants described 50 determinants to facilitate implementation that were mainly related to motivation due to positive experiences with AAT (Table 5). Examples of positive experiences with the dogs include the following:

P1 (female): “Contact with animals is often without labels, (pre) judgments, which makes it easier to receive new information and learn new skills. In addition, there is no problem with (un)professional physical distance to animals: you can have physical contact. Physical contact is in my point of view crucial for people (in need).”

P2 (female): “I am very happy with the practical aspects the therapy has brought me. I was pleased that it was very concrete, so I could perform the learned skills into my daily life. I also really liked that there was a lot of space to talk about things that happened in my daily life. I found the therapist very empathic and sympathetic, which I really appreciate. These 10 weeks may have given me more insight than 3 years of insight-based psychotherapy.”

The participants made additional suggestions for better implementation of AAT into mental health care. They suggested flexible time schedules for therapy sessions for participants who have a fulltime work schedule, multiple locations for therapy to reduce travel time and energy, and health insurance coverage to make the therapy accessible for people not otherwise able to participate in this research. Both the participants and therapists documented the importance of sharing information about the therapy and research results to inform people with ASD, health professionals, and stakeholders.

## 4. Discussion

The aim of this study was to explore the feasibility and relevance of, as well as barriers and facilitators to implementing, an animal-assisted therapy (AAT) in adults with ASD and to evaluate the credibility of the results of a previously conducted RCT.

With regard to reach, process evaluation may suggest selection bias with some consequences for external validity of the results. On the one hand, the process data showed that recruitment of participants was challenging: although potential participants responded positively to the information letter, only about 50% of approached patients enrolled in the study. On the other hand, females and dog owners seemed over-represented in our sample when compared with the worldwide population of females with ASD and dog owners within the Dutch population [22,23]. This could have influenced the external validity of the effect study.

Regarding the recruitment challenges, potential participants suggested limiting travel time to therapy locations, because this was the main reason the other 50% declined to participate in the study. Sensory sensitivity in people with ASD can make traveling a burden, and this aspect is relevant for other therapies as well. Offering a therapy at multiple locations might be a solution, but offering AAT at multiple locations poses logistical challenges: several conditions, such as suitable therapy rooms and eligible therapy dogs and therapists, must be met in order to perform AAT. Regarding the under-representation of male participants and non-dog-owners, the effect analyses were controlled for gender and dog ownership without influencing the estimated effects [10]. Nevertheless, future research on this topic should consider that selection bias might occur in this population, because males might not be reached easily. It is important to gain more insight into the effects of AAT in our under-represented groups and to explore whether larger sample sizes show the same results and effects when controlled for gender and dog ownership. Professionals in clinical practice and researchers need to improve information transfer about the intervention to attract more male patients and to involve those who do not own a dog.

Only two (external) factors were reported for missing a therapy session (i.e., participant illness and preplanned vacation); no motivational factors were reported. This seems to suggest that the participants were highly motivated to participate in research and receive AAT. This is also underlined by an adherence rate in the intervention condition, which was higher than in other studies in the adult ASD population [20,24]. All the participants attended at least nine out of 10 therapy sessions. It can be suggested that researchers and health professionals should focus more on providing information that attracts a larger proportion of those who might be interested in receiving the intervention than on improving adherence to the protocol, which seems a less important issue for those who are willing to follow the program.

Both the therapists and participants evaluated the intervention as feasible and relevant, and both reported being satisfied with the intervention program content. The participants were slightly more positive when appraising AAT for themselves than when appraising it for other people with ASD, which may reflect some inability to see the intervention from the perspective of others. A safe, joyful, and relaxing environment, the opportunity to touch another living being during therapy, concrete exercises, and direct feedback were cited by the participants and therapists as important determinants of their positive appraisal. These reported aspects are in line with the significant effects found in the effect study—namely, decreased self-reported stress and agoraphobia and increased proxy-reported social self-awareness and social communication [10]. The positive appraisal of touching the therapy dog is noteworthy, because many people with ASD experience sensory overstimulation when being touched by another person [25]. Physical contact with a therapy dog is possibly less overstimulating than physical contact with a human being. This may be related to difficulties in social communication experienced by people with ASD. Human–human interactions are often logically more complicated than human–animal interactions. It can be argued that the therapy dogs fulfill a need for proximity—a determinant that is challenging to fulfill in many therapist–participant therapies due to ethical restrictions and social–communicative impairments of the patient with ASD. Furthermore, the participants specifically reported AAT as being a joyful experience. The therapy might be experienced as less invasive when compared with other therapies.

Regarding program fidelity, no major deviations from the therapy protocol were reported. The literature shows that most interventions require substantial adjustments for people with ASD. For example, Spain et al. [11] reviewed six effect studies on cognitive behavioral therapy in adults with ASD. Although overall positive preliminary results were reported, Spain et al. recommended a prolonged assessment phase and an increased number of treatment sessions to ameliorate engagement with the therapist, enhance emotional literacy, and practice and improve generalization. The participants and therapists in our study suggested that the experience-based character of AAT is one of the main reasons for its high feasibility.

The role play exercise where the participant invents a character who interacts with the animal was evaluated as challenging. It is important to consider that this exercise might pose challenges for adults with ASD due to impairments in imagination and pretend play [26]. For future clinical execution of the therapy protocol, therapists may consider eliminating this exercise when a participant experiences difficulty in performing it.

This process evaluation revealed that potential barriers jeopardizing program fidelity might include aspects such as negative attitude towards therapy in general, stressful contextual factors in participants’ lives, and problems with generalization of learned behavior skills. Although these were reported by only four participants, health professionals should take these factors into consideration before starting the AAT program. Involving partners or other family members in the therapy process or assigning homework for the participants may help increase program fidelity.

In spite of AAT’s overall feasibility, optimal performance of the protocol requires several basic elements, such as well-trained, tested, and socialized dogs; certified health care professionals; knowledge of animal behavior by the health care professionals or animal handlers who are providing the AAT; and suitable therapy rooms with non-slippery floors. For these reasons, implementation of AAT might be more logistically challenging compared with therapies such as CBT. To facilitate implementation in the mental health care setting, it is important to provide information and share the positive effects of AAT. Practitioners should be aware that other health professionals or patients might not be used to having animals present in a clinical health care setting and may experience anxiety or have an aversion and/or allergy to animals.

Notably, none of the participants or therapists reported concerns about animal welfare (e.g., maximum number of working hours and relaxation for the therapy dogs) or hygiene (e.g., zoonosis) as barriers to implementation of AAT in clinical practice. Although studies on dog welfare during AAT sessions are very limited, Glenk’s recent review of animal welfare offers guidelines, such as the therapy dog’s familiarity with the therapist and environment and access to water and a quiet place to rest between sessions [27]. It is also important to note that not all animals are suited to be involved in therapy sessions. The International Association of Human Animal Interaction Organizations (IAHAIO) has produced a white paper providing guidelines for specific species that can be employed as therapy animals [17]. Furthermore, we highly recommend that each individual animal should be physically and mentally evaluated before being involved in a therapy program and have regular check-ups by an animal behavior expert or veterinarian to protect the well-being of the animal involved. Additionally, because the therapists in our therapy program were working in the role of both therapist and dog handler, it will be useful in future research to gain more insight into the barriers and facilitators to performing this dual role and into their experiences regarding monitoring the welfare of both the humans and animals involved in the therapy.

## 5. Conclusions

In our RCT on the effectiveness of dog-assisted therapy, results showed decreased self-reported stress and agoraphobia and improved proxy-reported social awareness and social communication in adults with ASD. The current process evaluation showed that besides the positive intervention effects, the intervention program was experienced as satisfying, feasible, and relevant for the target population by both the participants and therapists. Due to the positive intervention effects and the positive evaluation of the program, AAT can be considered a valuable addition to an existing (limited) treatment repertoire for adults with ASD. Improved self-insight, joy, relaxation, and physical contact were reported as important determinants of positive therapy appraisal. These determinants match with the study effects on reduction of self-perceived stress and agoraphobia and improved social awareness and social communication reported by proxies. To increase possible therapy effects, health professionals should consider abilities for generalization of learned skills and severity of contextual aspects. Additional efforts can be made to involve male patients and non-dog owners, and further research on these subgroups is welcomed.

To date, AAT interventions have not been regulated worldwide, and for this reason, it is very important when implementing AAT programs to consider the safety of all participants, including patients, health professionals, and animals. Sufficient education/training of therapists and animal handlers and animal welfare including veterinarian check-ups and regulated working hours must be carefully observed. Future research should focus on these factors, and professionalization of this field should include formulating international quality guidelines and certification of therapy animals and therapists.

## Figures and Tables

**Table 1:** Baseline characteristics.

Baseline Characteristic	Study Sample, N (%)	Overall Population, Ratio (%) *
Male	29 (55)	2:1 (33)
Dog owner	18 (34)	1:4 (20)
Age, groups (years)		
18–32	19 (36)	
33–46	16 (30)	
47–60	18 (34)	

* Loomes, Hull, and Mandy; Dibevo [22,23].

**Table 2:** Scores (Modus (25%/75%)) on the process evaluation questionnaire (PEQ).

Evaluated by	Satisfaction	Relevance for Themselves	Relevance for ASD	Feasibility for Themselves	Feasibility for ASD
Participants	4 (4/5)	4 (4/5)	4 (4/5)	4 (4/5)	4 (4/4)
Therapists	5 (5/5)	-	5 (5/5)	-	5 (5/5)

All variables were scored using the range from 1 (totally disagree) to 5 (totally agree). ASD = Autism Spectrum Disorder.

**Table 3:** Main reasons for deviation from the therapy protocol.

Factor	Reported Reason	N
Therapy protocol	Limited time	34
	Not able to (fully) perform the exercise	12
	The participant is already familiar with exercise	3
	Outside temperature above 25 °C	2
Therapy dog	Logistical reasons (volunteers not able to bring the dog)	5
	Rematch participant and therapy dog by therapist	4
	Illness dog	3
Therapist	Holiday therapist	6
	Illness therapist	3

The total number of participants was 51.

**Table 4:** Barriers to implementing the therapy protocol.

Label	Item Description	N
Basic requirements according to the participants	Lack of eligible therapy dogs	6
	Lack of suitable treatment rooms	4
	Lack of eligible therapists	2
Participant factors	Negative therapy attitude	2
	Stressful life events	1
	Problems with generalization of learned skills	1
Basic requirements according to the therapists	Lack of eligible therapy dogs	3
	Lack of suitable treatment rooms	3
	Lack of eligible therapists	3

The total number of participants was 26.

**Table 5:** Facilitators to implementing the therapy protocol.

Label	Item Description	N
Positive experiences according to the participants	Appraisal of the treatment as effective	19
	Practical and experience-based therapy	11
	Joy and feasibility of the therapy	8
	Feeling safe and relaxed	4
	Physical contact with dogs	4
	Variety and repetition of the exercises	3
	Possibility to share daily life problems with therapist/dog	2
Logistics	Flexible schedule of the sessions	2
	Multiple locations for therapy (distance)	1
Information/Communication	Sharing experiences with other participants/health professionals	3
	Sharing information on research outcomes	3
Insurance/Costs	Coverage by health insurance	1

The total number of participants was 26.

## Appendix A. PEQ Participant Version

**1. How satisfied are you with the therapy?**

**Table:**

1	2	3	4	5
Not satisfied at all	Not satisfied	Neutral	Satisfied	Very satisfied

**2. How relevant is this therapy for you?**

**Table:**

1	2	3	4	5
Not relevant at all	Not relevant	Neutral	Relevant	Very Relevant

**3. How relevant is this therapy for adults with ASD?**

**Table:**

1	2	3	4	5
Not relevant at all	Not relevant	Neutral	Relevant	Very relevant

**4. How feasible is this therapy for you?**

**Table:**

1	2	3	4	5
Not feasible at all	Not feasible	Neutral	Feasible	Very feasible

**5. How feasible is this therapy for adults with ASD?**

**Table:**

1	2	3	4	5
Not feasible at all	Not feasible	Neutral	Feasible	Very feasible

**6. How many AAT sessions did you complete?**

**7. Which aspects/parts of the intervention do you think are barriers to implementing AAT (implementation means: introduction of this therapy) within a mental health care center?**

**8. Which aspects/parts of the intervention do you think are facilitators to implementing AAT (implementation means: introduction of this therapy) within a mental health care center?**

**9. Additional comments**

## Appendix B. PEQ Therapist Version

**1. How satisfied are you with the therapy?**

**Table:**

1	2	3	4	5
Not satisfied at all	Not satisfied	Neutral	Satisfied	Very satisfied

**2. How relevant is this therapy for adults with ASD?**

**Table:**

1	2	3	4	5
Not relevant at all	Not relevant	Neutral	Relevant	Very relevant

**3. What do you think is relevant/not relevant in this therapy?**

**4. How feasible is this therapy for adults with ASD?**

**Table:**

1	2	3	4	5
Not feasible at all	Not Feasible	Neutral	Feasible	Very feasible

**5. What do you think is feasible/not feasible in this therapy?**

**6. Which aspects/parts of the intervention do you think are barriers to implementing AAT (implementation means: introduction of this therapy) within a mental health care center?**

**7. Which aspects/parts of the intervention do you think are facilitators to implementing AAT (implementation means: introduction of this therapy) within a mental health care center?**

**8. Additional comments**
